# Two Interesting Cases of Skin Disease

**Published:** 1888-07

**Authors:** Elbert Wing

**Affiliations:** Demonstrator of Pathology at Chicago Medical College; 3266 Cottage Grove Avenue


### The Medical Journal and Examiner.

VOLUME LVII. JULY, 1888. NUMBER 1.

### ORIGINAL COMMUNICATIONS.

### REPORT OF TWO INTERESTING CASES OF SKIN DISEASE: I. URTICARIA CHRONICA RECURRENS. II. DERMATITIS MEDICAMENTOSA.

BY ELBERT WING, A. M., M. D.,*
Demonstrator of Pathology at Chicago Medical College.

I.—CHRONIC RECURRENT URTICARIA.

The patient, a widow 37 years of age, was employed as housekeeper in one of the public institutions of this city when her case came under observation, on March 8, 1888. For the sake of brevity only those facts in the family history which have an evident relation to this case are reported.

The patient's father died when she was a child, but she knows, however, that he was subject to attacks which resemble her own, and that in his case the throat was at times the site of the lesion. She also knows that one of these attacks in which the throat was involved proved fatal. Her mother is 66 years old, and except occasional attacks, during the last few years, of what is called "neuralgia of the stomach and bowels," she has enjoyed excellent health. Two of her brothers have suffered with the same localized swellings for which she now seeks relief. One of them has been a much greater sufferer than she herself, and the other has had much lighter attacks. One brother and one sister have been free from the trouble.

In all cases in which the disease has occurred in the family it has frequently recurred, and has been obstinately chronic. The essential clinical features have been so similar in all of the cases that a description of this patient's case will answer for the others.

In regard to her health during childhood and adolesence, she says that she has had no complaint except the localized swellings. These, however, began as early in her life as she can remember, and she has never been free from them for any considerable period of time. Although closely and repeatedly questioned in regard to gastric troubles, she insists that she had none before her twentieth year.

Since that time, however, she has been troubled with gastric flatulence, which is at times very moderate, and others extreme. This begins soon after meals, and is more marked after the injestion of certain kinds of food. Uncooked apples produce the greatest disturbance, and even the smallest piece will bring on an attack. Associated with this flatulence there is regurgitation of both sour fluids and of food. The regurgitated food is always intensely sour to the taste, and it comes up in the throat at all hours after eating. She says that she has repeatedly detected food in

*Read before the Chicago Medical Society, July 2, 1888.

this way, which had been eaten twenty-four hours before its regurgitation.

For years her bowels have been obstinately constipated. She has the often vicious habit of waiting for a prompting, and frequently passes three days without defecation. She is not troubled with intestinal flatulence.

Menstruation began when she was thirteen and one-half years old and has presented no markedly unusual features. She was married in her twentieth year, has one son fifteen years old and has had no miscarriages. She, however, complains of an aching sensation in the sacral region, which is rather constantly present and always worse during the catamenial period. Two years ago she was told by a physician that her uterus was retroverted, and at his suggestion wore an internal pessary which she thinks diminished this sacral pain. She was, however, treated at the same time for stomach troubles, also with benefit.

She has a moderate amount of intermittent cutting pain in the left ovarian region, but has not observed that it bears any relation to her menstrual periods. She takes cold easily, has severe so-called head-colds, with a free secretion from the nasal and bronchial mucous membranes, but at other times is free from cough.

She is occasionally troubled with what she calles "a humor" on the inner surface of the thighs, but it is not now present, and she is not aware that it bears any relation to her other troubles.

What, in the opinion of the patient, constitutes her ailment, and what she seeks relief for is the occurrence of the localized swellings and their associated gastric disturbances.

The first symptom of one of these attacks is a sense of numbness and tingling in the part of the surface involved. The skin in this area begins to feel thick and hard, and it is swollen and moderately and uniformly red and hot. It becomes moderately œdematous, the itching and pain on pressure are very slight, and there are no white or yellowish-white areas over these tumor-like swellings at any time. When the area involved is easily inspected by the patient, as upon the extremities, she has noticed a streak of somewhat brighter red than that of the general surface which passes across it in the course of the lymphatics. This always terminates at the edges of the swelling.

The tumefaction proceeds steadily to the maximum, which is usually reached in twenty-four hours, and it is rather longer in disappearing than in developing. In all attacks the swelling is very marked, and in some its limit seems to be only that of the possible distensibility of the skin, which is frequently left covered with large blebs when the swelling subsides. For example, the hands become so swollen that the fingers are extended and widely separated, and the tips of the thumb and little finger cannot be brought into apposition. The face swells until the eyes are completely closed, and when the arms are attacked, sleeves which ordinarily are loose cannot be drawn over them. When the swelling begins upon the upper or fore arm it rarely extends beyond the elbow.

Any part of the body, or several parts at the same time, may be attacked, as both feet and ankles, or one foot and one hand. In the latter case a foot and hand of the same side are usually attacked. The patient has had mild, but never very severe, attacks in the pharynx.

She has a chronic gastric catarrh of mild severity, which, at the time of these attacks, is sometimes subject to violent exacerbations. So far as she has observed, the localized swellings precede the gastric symptoms. In severe attacks there is very great nausea with frequent emesis, and sharp colicky pain in the stomach. At these times the stomach is absolutely intolerant of both liquids and solids, and the vomited matters are intensely sour. This gastric intolerance continues, after severe paroxysms for several days. For a number of years the colicky pains in the stomach have grown less severe. There is no intestinal disturbance at all in connection with these attacks.

While she is usually a light sleeper, during these attacks the patient is exceedingly somnolent and complains of great lassitude.

Until recent years the attacks have invariably occurred with the menstrual periods, but they have always, and do now occur at other times. Fatigue following hard physical exertion has always been a sure forerunner of an attack, and any work which requires much stooping is certain to bring one on. A smart blow upon any part of the body will produce at the point of traumatism a swelling which runs the usual course, without, however, the associated stomach troubles. She has never observed the phenomenon which gives rise to the term *urticaria factitia.*

Physical examination reveals a woman of strong, robust appearance, considerable adipose tissue, florid face, and fair hair. There is a mild subacute nasal catarrh present. The heart is slightly displaced upward. Sounds are normal. In the left infrascapular region there is some interrupted respiration, and the vesicular murmur over this entire lung is slightly less than over the other one. The stomach is distended with gas, but there is no evidence of gastric ectasia. The ascending and transverse divisions of the colon are moderately distended with gas.

The uterus is possibly retroverted a very little, is of the normal depth, and freely movable without pain. There is no tenderness at all about the pelvic viscera.

An examination of the urine was not thought necessary, and was not made.

The patient was told that the gastric catarrh was probably the cause of her troubles, and a hopeful prognosis given her.

The customary advice in regard to diet and the movements of the bowels was forcibly presented to her, and she was advised to take five drops of the dilute hydrochloric acid fifteen minutes before meals, three times daily, and to increase the dose gradually to ten drops. She was also requested to take Johnson & Johnson's papoid and soda tablets, one immediately after, and one more one hour after eating, three times daily. These tablets each contain one grain of papoid and two of bicarbonate of sodium.

On June 15, fourteen weeks after the first consultation, the patient was seen in an attack, the first of any severity since she began treatment. She says that for a week she has been acting as cook for the institution. A few days after she began cooking she began to have what she calls warning attacks of moderate swellings. To-day these have culminated. Both forearms and hands are very greatly swollen, but only very slightly, and on very deep and firm pressure, œdematous. The swollen surface is only moderately red, and the burning and itching is very slight. The stomach is moderately deranged. Menstruation is not present.

The patient says that since she began the treatment mentioned, until she began cooking, she was free from the local swellings, and also from the gastric troubles. The diagnosis seems reasonably certain, since, although the swellings resemble the erythemas in some respects, they very closely correspond with those in the case reported by Milton, and designated by Kapasi, as giant urticaria (Riesenurticaria). (Kapasi's P. u. T. der Hautkrankheiten, 2te Auflage, s. 307.)

II.—Dermatitis medicamentosa.

The patient in this case is a professor of Greek in one of the colleges of this State, and sends his own description of the case. It is graphic enough for the purpose of diagnosis, and is submitted in his own language.

He writes, "When otherwise in my usual excellent health, I took cold this week, and upon the doctor's prescription took one two-grain cinchonidia pill. In three hours my knuckles and hands swelled up, and for forty-eight hours they itched and burned most intolerably, making sleep impossible during the first night. This is the third time within a year that this result has followed a dose of quinine or cinchonidia in my case, and I have not taken these drugs at other times."

The authors of works on diseases of the skin classify these cases somewhat differently. Thus, Eichhorst (Pathologie und Therapie, 1te Auflage, s. 230) refers to them under the heading *Erythema exudativum toxicum.* Kapasi (P. und T. der Hautkrankheiten, s. 310) places them among the urticarias. Hyde (Diseases of the Skin, 2d edition, p. 184) adopts the classification of the American Dermotalogical Association, which is the one employed in this report, and refers to Morrow's report of sixty cases (*N. Y. Med. Jour.*, March, 1880, p. 244) of dermatitis caused by the ingestion of cinchona and its alkaloids. He says, "The prevailing type is erythematous, the rash being a bright vivid hue, disappearing on pressure and resembling scarlatina."

Hyde and Eichhorst call attention to the fact that frequently in these cases the quantity of the article taken bears no relation to the violence of the clinical symptoms, and Kapasi says that experiments show that the results are independent of a knowledge, on the part of the patient, of the ingestion of the offending substance.

3266 Cottage Grove Avenue.

